# Evanescent-Wave Fiber Optic Sensing of the Anionic Dye Uranine Based on Ion Association Extraction

**DOI:** 10.3390/s20102796

**Published:** 2020-05-14

**Authors:** Takuya Okazaki, Tomoaki Watanabe, Hideki Kuramitz

**Affiliations:** 1 Department of Environmental Biology and Chemistry, Graduate School of Science and Engineering for Research, University of Toyama, Toyama 930-8555, Japan; kuramitz@sci.u-toyama.ac.jp; 2 Department of Applied Chemistry, School of Science and Technology, Meiji University, Kanagawa 214-8571, Japan; tomowata@meiji.ac.jp

**Keywords:** evanescent-wave fiber optic sensor, ion association, uranine

## Abstract

Herein, we propose an evanescent-wave fiber optic sensing technique for the anionic dye uranine based on ion association extraction. The sensor was prepared by removing a section of the cladding from a multimode fiber and hydrophobization of the exposed core surface. Uranine was extracted in association along with hexadecyltrimethylammonium (CTA) ion onto the fiber surface and detected via absorption of the evanescent wave generated on the surface of the exposed fiber core. The effect of CTA^+^ concentration added for ion association was investigated, revealing that the absorbance of uranine increased with increasing CTA^+^ concentration. A change in the sensor response as a function of the added uranine concentration was clearly observed. The extraction data were analyzed using a distribution equilibrium model and a Freundlich isotherm. The uranine concentration in the evanescent field of the fiber optic was up to 54 times higher than that in the bulk solution, and the limit of detection (3σ) for uranine was found to be 1.3 nM.

## 1. Introduction

Evanescent field spectroscopy using optical waveguides is an extension of the established spectroscopic technique attenuated total reflection (ATR) spectroscopy. During the last two decades, fiber optics with exposed cores have been applied for the ATR technique [1,2,3,4,5,6]. This technique is based on the presence of an evanescent wave generated in the exposed fiber core when the light traveling in the fiber core undergoes total internal reflection. If any absorbing molecules exist within the evanescent field, the evanescent wave is absorbed and results in attenuation in the amplitude of the propagating wave guided in the fiber core [7]. This phenomenon has been applied to chemosensors and biosensors for numerous analytes, including oxygen, H^+^ ions (i.e., pH), nitrates, silica, fluorides, melamine, DNA, and *Escherichia. coli* [8,9,10,11,12,13,14]. Evanescent-wave fiber optic sensors offer a number of advantages, such as high sensitivity, low cost, simplicity, and device convenience in terms of size and portability, allowing real-time multiple-location remote monitoring. In addition, the optical signals of such sensors are not affected by solids suspended in the sample solution because the sensing interactions only occur in the evanescent field, which extends only approximately 1 µm from the fiber surface [15].

Previously, we have developed exposed core fiber optic sensors for ATR-based determination of electrochemical species, geothermal scale, biofilms, and anionic surfactants [16,17,18,19]. For instance, the sensor for anionic surfactants can detect dodecyl sulfate at the mg/L level using the cationic dye ethyl violet which strongly adsorbs the fiber core because of the negatively charged hydroxyl groups on SiO_2_ [19]. However, a limitation of these sensors is their low sensitivity to anionic species, which is because such sensors work on the basis of the adsorption of analytes onto the negative fiber surface. Coating the fiber with a positively charged self-assembled monolayer has been demonstrated to improve the adsorption of Fe(CN)_6_^3−^ on the fibers [17]. However, this required a complicated layer-by-layer coating process and cleaning after each use.

Ion association extraction is a technique used for the extraction of analytes based on their association with an amphiphilic counter ion [20]. This technique has been combined with solvent extraction or solid-phase extraction for highly sensitive and/or selective detection of numerous chemical analytes [21]. Indeed, the extraction for anionic surfactants using ion association has been adopted as an official analytical method in Japan [22]. Recently, extraction into a phase composed entirely of an ionic associate species has been utilized for spectrophotometry, HPLC, and graphite furnace atomic absorption spectrometry [23,24,25].

Taguchi et al. have studied the sorption of ion associate agents onto membrane filters as a homogeneous phase by assuming that the membrane filter behaves like an organic solvent [26]. The extraction constants were investigated using organic ions having different alkyl chain lengths and membrane filters made of different materials.

Uranine, which is the disodium salt of the xanthene dye fluorescein and is used as a model anionic dye analyte in this study, is widely used as a coloring additive in drugs and cosmetics [27,28]. Uranine exhibits strong fluorescence properties for optical and bio sensing. The toxicity of uranine (in combination with phloxine B) to the Mediterranean fruit fly has been used to control its numbers in coffee fields [29]. Although apparently not pesticidal on its own, it has been found to synergize the toxicity of Rose Bengal to mosquito larvae, possibly by extending the range of light wavelengths from which it can absorb and utilize energy [30]. Uranine is also used as a fluorescent tracer in geothermal and hydrospheric research. For instance, uranine has been used to investigate the fates of thermal aquifers injected into geothermal wells [31]. These tests typically involve the injection of several kilograms of uranine. 

Foguel et al., reported an evanescent wave fiber optic sensor for detection of Basic Red 9 dye [32]. The sensor comprised a polystyrene fiber optic coated with a molecularly imprinted polymer that was synthesized using 2-crylamido-2-methyl1-propanesulfonic acid and ethylene glycol dimethacrylate as a functional monomer and crosslinker, respectively. The sensor was demonstrated to be low cost, selective, sensitive, simple to manufacture, and disposable.

In this study, we have developed a sensor for uranine that combines evanescent-wave fiber optics and ion association extraction. Hexadecyltrimethylammonium is used as a cationic surfactant, and detection is based on the evanescent wave generated at the surface of the exposed fiber core, which is hydrophobized via the formation of siloxane bonds using Sigmacote. The ion extraction data were analyzed using an equilibrium distribution model and an adsorption model. To the best of our knowledge, this is the first report of evanescent-wave fiber optic sensing combined with ion association extraction for an anionic dye.

## 2. Material and Methods

Uranine and acetone were purchased from Wako Pure Chemical Industries, Ltd. Hexadecyltrimethylammonium bromide (CTAB) was obtained from Tokyo Chemical Industry Co., Ltd. Solutions were prepared by dissolving the compounds in Milli-Q water. An FT200EMT (Thorlabs, USA) step index multimode optical fiber with a 200 μm-diameter fused-silica core was used. Acetone was used to remove the plastic cladding from an 8 cm section of the fiber in order to expose fiber core surface, which was used as the sensing area. The fiber sensor was connected to a white-light source (ELI-050J-OPT3077; Mitsubishi Rayon, Japan) and a spectroscopic detector (MV-3100; Jasco, Japan), as shown in Figure S1. 

Sigmacote^®^ obtained from Sigma-Aldrich, Inc., was used to form a hydrophobic film on the fiber core [33]. The fiber was immersed in Sigmacote and then washed with water in accordance with the manufacturer’s instructions [34]. The fiber optic sensor was placed on a watch glass, and the absorbance measurements were performed by immersing the fiber core in the sample. Sensor absorbance values, defined as *A* = −log (*I*/*I*_0_), were acquired by recording the light intensity through the fiber in water (*I*_0_) and the intensity upon exposure to the sample (*I*). In this study, the Sigmacote coating was stable for 6 days.

For validation and comparison, the absorbance of solutions was also measured in 1 cm cuvettes using a conventional spectrophotometer (UV-2450; Shimadzu, Japan).

## 3. Results and Discussion

### 3.1. Optimization of CATB Concentration

**Figure 1:**
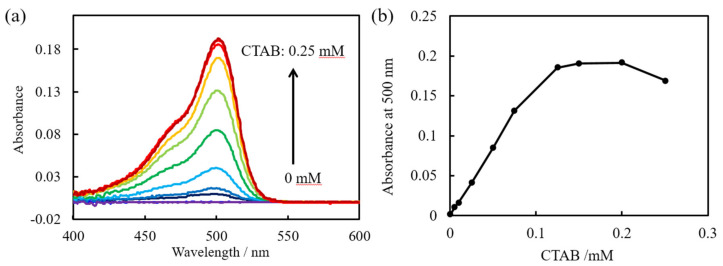
(**a**) Absorbance spectra of various concentrations of hexadecyltrimethylammonium bromide (CTAB) and 1 µM uranine obtained 300 s after mixing by the fiber optic sensor prepared in this study. (**b**) Absorbance at 500 nm as a function of CTAB concentration.

Figure 1a shows the absorbance spectra recorded 5 min after mixing 1 µM uranine and CTAB at different concentrations as measured by the fiber optic sensor. For 0 mM uranine, no absorbance spectrum is detected. With increasing CTAB concentration, the absorbance of uranine is more clearly observed. The shapes of the absorbance spectra are similar to those measured using a conventional spectrophotometer. Figure 1b shows the change in absorbance at 500 nm vs. CTAB concentration. The absorbance increases with increasing concentration from 0 to 0.125 mM before plateauing. Thus, uranine in the sample solution can be detected with the fiber optic sensor upon the addition of CTAB. CTA (B) is a univalent cationic surfactant that is widely used as a cation to form ion association complexes with target anions [20]. In order to form an ion association complex and adsorb onto the fiber within 5 min, CTAB at a concentration of 125 µM, which is 125 times that of uranine, is required.

### 3.2. Distribution Equilibrium Model

In this experiment, CTA (B) and uranine formed an ionic association complex that adsorbed onto the hydrophobized surface of the fiber optic core. The hydrophobic and uncharged complex formed distributes on the surface of the hydrophobic fiber core, which is similar in principle to solvent extraction of hydrophobic ion pairs. Therefore, the distribution equilibrium for the ion association complex between the aqueous phase and the fiber surface can be interpreted in the same terms as those used for solvent extraction [26]. If we ignore the dissociation and polymerization of the ion association phase at low concentration, the distribution of the ion associate can be described as follows:

2CTA^+^ + uranine^2−^ ⇌ {(CTA^+^)_2_ · uranine^2−^}_aq_ ; *K*_f_ = [(CTA^+^)_2_ · uranine^2−^]_aq_ / [CTA^+^]^2^ [uranine^2−^] 
{(CTA^+^)_2_ · uranine^2−^}_aq_ ⇌ {(CTA^+^)_2_ · uranine^2−^}_fiber_ ; (1)

*K*_d_*=* [(CTA^+^)_2_ · uranine^2−^]_fiber_/ [(CTA^+^)_2_ · uranine^2−^]_aq_ (2)

*K*_ex_ = [(CTA^+^)_2_ · uranine^2−^]_fiber_ / [CTA^+^]^2^ [uranine^2−^] = *K*_f_*K*_d _ (3)

where *K*_f_, *K*_d_, and *K*_ex_ are the formation, distribution, and extraction constants, respectively. If it is assumed that the concentrations of the ion associate ([(CTA^+^)_2_ · uranine ^2−^]_aq_) in water much lower than that on the fiber ([(CTA^+^)_2_ · uranine ^2−^]_fiber_), the distribution ratio *D* for uranine is given by

*D* = [(CTA^+^)_2_ · uranine^2−^]_fiber_/{[uranine^2−^] (4)

By combining Equations (3) and (4), we obtain

*D* = *K*_ex_ [CTA^+^]^2^ (5)

indicating that *D* is proportional to the square of [CTA^+^].

**Figure 2:**
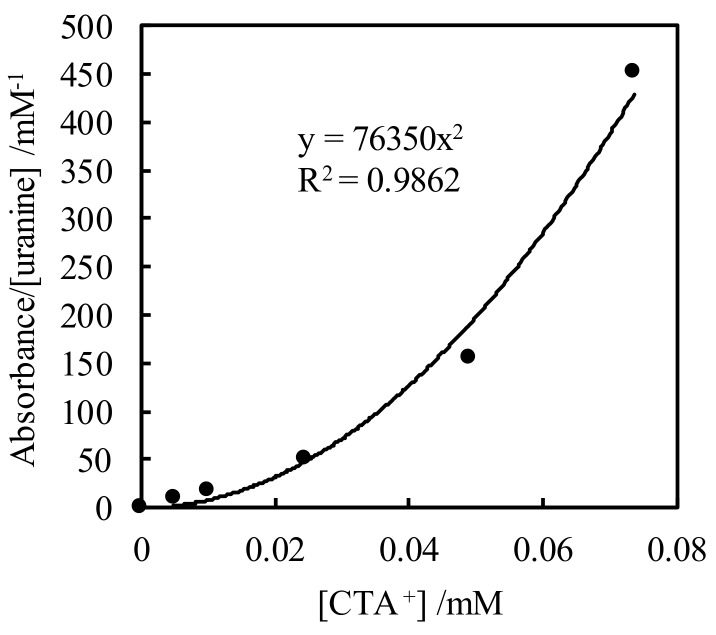
Plot of absorbance/[uranine] vs. the equilibrium concentration of CTA^+^.

Figure 2 shows the relationship between absorbance and [CTA^+^]. The equilibrium concentrations of CTA^+^ and uranine^2−^ were calculated from absorbance assuming that the uranine in the water is completely extracted onto the fiber surface in the concentration range where the absorbance is saturated. The absorbance obtained from a fiber optic sensor correlates linearly with the concentration of the analyte in the evanescent field. A quadratic relationship is observed between absorbance/[uranine^2−^] and [CTA^+^]. This shows that the adsorption of the uranine–CTA^+^ complex onto the fiber surface can be explained by an equilibrium model in a similar way as solvent extraction. However, for a more accurate model of extraction by the sensor, other factors must be considered, including the time to reach absorbance equilibrium, and the accurate determination of the ion associate on the fiber and the uranine and CTA^+^ in the solution.

### 3.3. Adsorption Model

**Figure 3:**
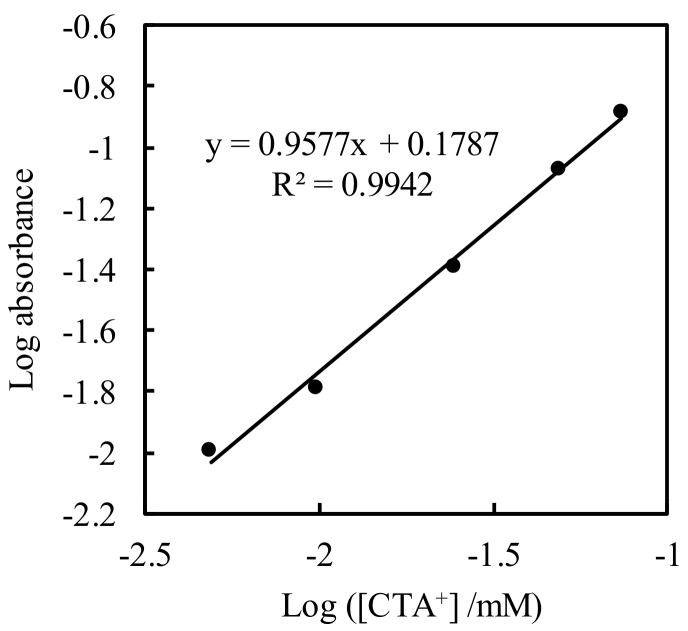
Plot of log absorbance versus log equilibrium concentration of CTA^+^.

As an alternative to the equilibrium model, the experimental results can be interpreted using a Freundlich adsorption model. The Freundlich isotherm is an empirical equation for interpreting multilayer adsorption. It is given as follows:

(6) $$W=K_{F}C^{1/n}$$

where *W* is adsorbed amount, *K_F_* and 1/*n* are adsorption constants, and *C* is equilibrium concentration. Thus, adsorption following a Freundlich isotherm shows a linear relationship between logarithmic *W* and *C*. The isotherm is shown in Figure 3, where the absorbance obtained by the fiber sensor indicates adsorbed amount: *W*. A linear relationship (defined as y = 1.5091X^0.9577^) is obtained, and the 1/*n* value is 0.9577 (*K_F_* in this figure has no meaning because *W* is not the actual adsorbed amount). The 1/*n* value between 0 and 1 is associated with a chemisorption process [35]. 

The Langmuir isotherm, which supposes monolayer adsorption, does not fit well to the experimental data (R^2^ = 0.79). In a previous study, the adsorption between a negatively charged fiber sensor and positively charged ethyl violet was found to follow the Langmuir isotherm [19]. This difference was because adsorption of the ion association complex was not saturated.

### 3.4. Regeneration of the Fiber Sensor

**Figure 4:**
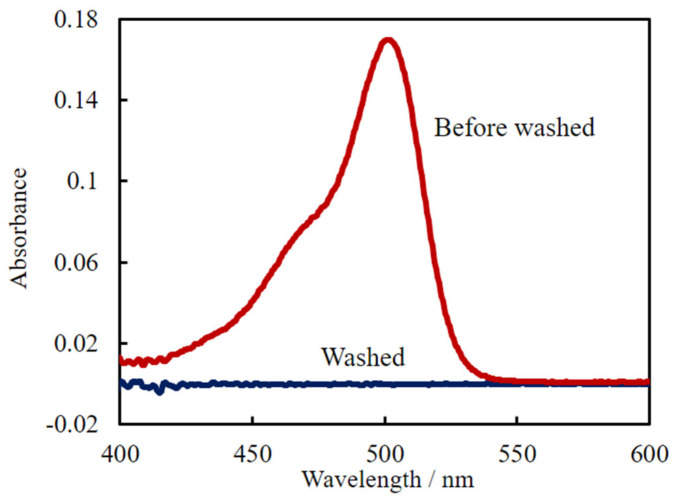
Absorbance spectrum of 1 µM uranine/0.25 mM CTAB obtained using the fiber optic probe and that after washing with water.

Regeneration is an important factor for usability in sensors. In many cases, sensors with strongly sorbed analytes need to be regenerated prior to each use by complicated techniques involving chemicals, such as acids, bases, oxidants, and reductants, or irradiation by UV light or plasma [36]. In previous work on our fiber optic sensor, the dyes on the fiber were removed by an acetone wash [17,18,19]. Following analysis of dyes at high concentrations, the fiber must be wiped clean with a Kimwipe soaked in acetone (very carefully to avoid breaking the quartz fiber). This is complicated and requires the use of an organic solvent and remeasurement of the reference intensity for each use. However, in the present study, the CTA–uranine associate on the fiber sensor could be washed off with water alone. Figure 4 shows the absorbance spectra from the sensor before and after washing with pure water. In the absorbance spectrum measured after washing, no signal due to the ion associate is observed. Thus, all measurements were easily performed by washing with pure water before and after use. This is due to the polarity of CTA^+^ as a surfactant, imparting solubility in water.

### 3.5. Determination of Uranine

**Figure 5:**
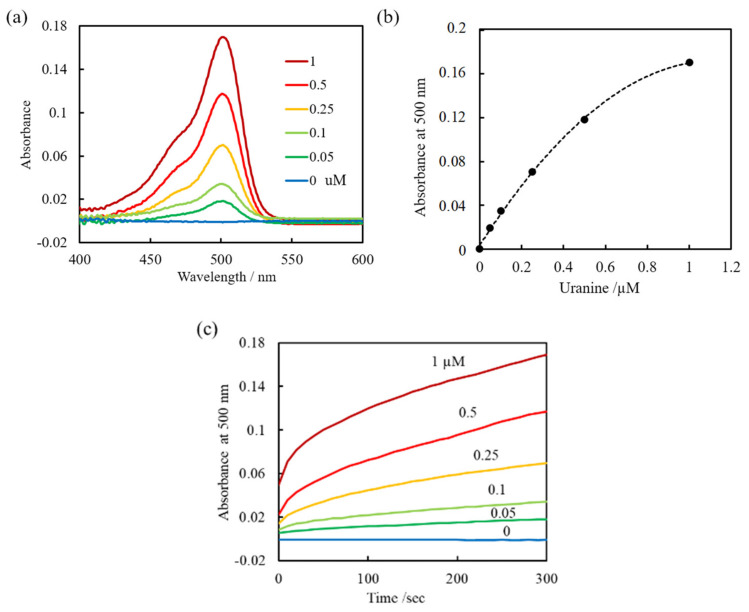
(**a**) Absorbance spectra obtained by the fiber optic sensor 300 s after mixing uranine at various concentrations and 0.25 mM CTAB. (**b**) Absorbance at 500 nm as a function of uranine concentration. (**c**) Absorbance at 500 nm as a function of time.

Figure 5a and b show the changes in absorbance and at 500 nm as a function of the initial concentration of uranine with 0.25 mM CTAB. The absorbance intensity clearly increases with increasing uranine concentration. Figure 5c shows the changes in absorbance as a function of time after immersion in sample solutions. The absorbance did not reach equilibrium in 5 min. However, the differences in absorbance with the concentration of uranine were clearly observed at all times. The gradient of the curve for the absorbance at 500 nm vs. concentration decreases with increasing concentration. Unlike the results shown in Figure 1, when the concentration of the counter ion is too high (CTA^+^; 0.25 mM) the effect on the distribution of excess ion association complex in the aqueous phase is not negligible. It is assumed that the complex in the linear range of concentration from 0 to 0.1 µM (R^2^ = 0.9974) is completely distributed on the fiber. Uranine remaining in the bulk solution was calculated from the slope of the line, for example, it is approximately 50% for the 1 µM sample. Figure S2 shows the relationship between [(CTA^+^)_2_ · uranine^2−^]_aq_ and absorbance, which exhibits linear correlation with [(CTA^+^)_2_ · uranine^2−^]_fiber_. These should be linear according to Equation (2), but the linearity is slightly lower. This may be attributed to the weaker hydrophobic interaction between ion associates than that between the fiber surface and the ion associates. Therefore, the distribution equilibrium model should be applied for low concentrations of ion associate.

With the fiber optic sensor, an evanescent wave at the surface of the core penetrates the surrounding medium to a limited depth. The equation for evanescent wave absorption based on Beer’s law is given by

(7) $$A=4\frac{\sqrt{2}}{3}\frac{\lambda }{2\pi r\sqrt{n_{core}^{2}-n_{cladd}^{2}}}\frac{\alpha CL}{2.303}$$

where *C* is the concentration, *λ* is the wavelength, *α* is the absorption coefficient, *r* is the radius of the fiber core (100 µm in this experiment), *L* is the length of the exposed fiber core (8 cm in this experiment) [7], and *n_core_* and *n_cladd_* are the refractive indices of the core and cladding at 500 nm (calculated to be 1.464 and 1.428 from the values at 436 and 589.3 nm in the data sheet supplied with the FT200EMT fiber, respectively [37]). The equation indicates linear relationships between the absorbance of the sensor and the absorption coefficient of the analyte, concentration, and exposed core length. Using Equation (7), the absorption coefficient was obtained to be 21,200 × 10^3^ M^−1^ cm^−1^ from the slope in the linear region from 0 to 0.1 µM in Figure 5b. However, the absorption coefficient in uranine solution was measured to be 395,000 M^−1^ cm^−1^ using a conventional spectrophotometer. This disagreement shows that the concentration of uranine in the evanescent field at the fiber sensor surface is up to 54 times higher than that of the bulk solution in the linear concentration range. The limit of detection calculated as 3σ/slope in the linear region (where σ denotes the standard deviation of the blank measures) was thus found to be 1.3 nM, which is higher than that achieved with conventional spectrophotometry. The relative standard deviations of samples were in the range of 1.1–1.9 % (n = 3). From Equation (7), the sensitivity of the absorbance using the fiber optic sensing proportionally increases with increasing exposed core length. In this study, a core length of 8 cm was adopted because 200 µm diameter fused-silica core is fragile [15]. This technique is based on the addition of CTA. Therefore, anionic hydrophobic molecules, such as anionic surfactants with high concentration at mM level consuming added CTA, will reduce the sensor absorbance.

Furthermore, as demonstrated by the data in Figure 1b, the sensor can also be used to measure the concentration of a cationic surfactant in the presence of uranine at a high and constant concentration.

## 4. Conclusions

We presented an evanescent-wave fiber optic sensing technique for the cationic dye uranine based on ion association extraction. Uranine was concentrated on the fiber surface as an ion associate with CTA^+^ and its absorbance was measured from the attenuation of an evanescent wave generated on the fiber. The distribution and adsorption of the ion associate were analyzed using a distribution equilibrium model and the Freundlich isotherm. The distribution equilibrium model accurately described the effect of an ion associate with a stoichiometry of 2 mol CTA^+^ and 1 mol uranine^2−^. The concentration of the uranine in the evanescent field of the fiber optic was found to be up to 54 times than that in bulk solution. Thus, highly sensitive uranine determination with a limit of detection (3σ) of 1.3 nM was achieved.

This sensing technique has many advantages in terms of its high sensitivity, convenience, low cost, portability, and low sample volume. We have elsewhere developed a U-shaped fiber optic sensor [38], allowing many samples in small tubes to be measured rapidly by inserting the sensor, which can be easily washed between readings with water only. This sensing technique is applicable to the determination of various ionic analytes in addition to just dyes. Moreover, the absorbance of cations can be measured using an anionic surfactant as the counter ion, which will be analyzed by the distribution equilibrium model in future work.

## Supplementary Materials

The following are available online at https://www.mdpi.com/1424-8220/20/10/2796/s1, Figure S1: Figure S1. Plot of absorbance vs. [(CTA ^+^)_2_ uranine ^2−^]_aq_; Figure S2. Plot of absorbance vs. [(CTA ^+^)_2_ uranine ^2−^]_aq_.
