# Supplementary material for: Evanescent-Wave Fiber Optic Sensing of the Anionic Dye Uranine Based on Ion Association Extraction
Source: Sensors (Basel). 2020 May 14;20(10):2796. doi: 10.3390/s20102796 (PMC7287843; doi:10.3390/s20102796)
Supplement: Supplementary file 1 [file sensors-20-02796-s001.pdf]

Supplementary Materials

Evanescent-wave fiber optic sensing of the anionic dye uranine based on ion association extraction

Takuya Okazaki [1,2]*, Tomoaki Watanabe [2], and Hideki Kuramitz[2]

[1] Department of Environmental Biology and Chemistry, Graduate School of Science and Engineering for Research, University of Toyama, 3190 Gofuku, Toyama 930-8555, Japan.
[2] Department of Applied Chemistry, School of Science and Technology, Meiji University, 1-1-1, Higashimita, Tama-ku, Kawasaki, Kanagawa 214-8571, Japan.
* Correspondence: okazaki@meiji.ac.jp; Tel.: +81-44-934-7224

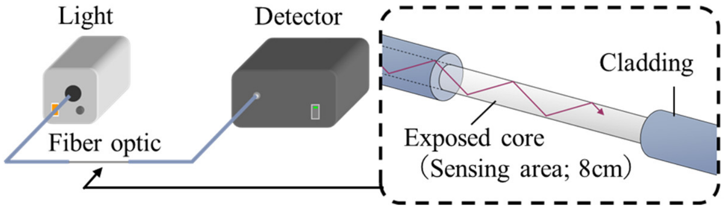

Figure S1. Schematic diagram of the experimental setup.

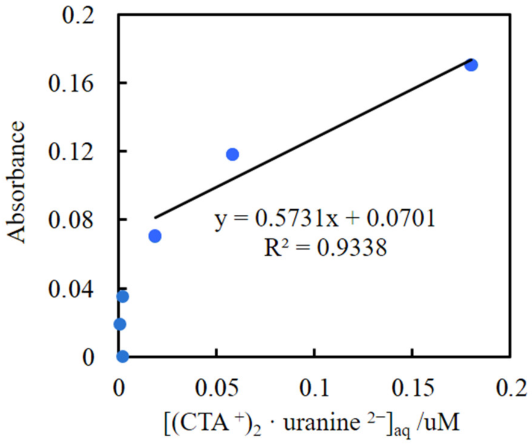

Figure S2. Plot of abrobance vs. $[(CTA^{+})_2 \cdot uranine^{2-}]_{aq}$
